# Illness perceptions; exploring mediators and/or moderators in disabling persistent low back pain. Multiple baseline single-case experimental design

**DOI:** 10.1186/s12891-022-05031-3

**Published:** 2022-02-11

**Authors:** E. J. Edwin de Raaij, H. Harriet Wittink, J. F. Francois Maissan, J. Jos Twisk, R. W. J. G. Raymond Ostelo

**Affiliations:** 1 Research Group Lifestyle and Health, University of Applied Sciences Utrecht, Heidelberglaan 7, 3584 CS Utrecht, The Netherlands; 2 Department of Health Sciences, VU University, The Netherlands and the EMGO Institute for Health and Care Research, Amsterdam, The Netherlands; 3 Department of Clinical Epidemiology and Biostatistics, VU University Medical Centre, Amsterdam, The Netherlands; 4 Department of Health Sciences, Faculty of Science, Vrije University Amsterdam, Amsterdam, the Netherlands; 5 Department of Epidemiology and Data Science, Amsterdam UMC, Vrije Universiteit Amsterdam, Amsterdam Movement Sciences Research Institute, Amsterdam, the Netherlands

**Keywords:** Low back pain, Illness perceptions, Mediation, Moderation, SCED-study, Physiotherapy

## Abstract

**Introduction:**

Illness Perceptions (IPs) may play a role in the management of persistent low back pain. The mediation and/or moderation effect of IPs on primary outcomes in physiotherapy treatment is unknown.

**Methods:**

A multiple single-case experimental design, using a matched care physiotherapy intervention, with three phases (phases A-B-A’) was used including a 3 month follow up (phase A’). Primary outcomes: pain intensity, physical functioning and pain interference in daily life. Analyzes: linear mixed models, adjusted for fear of movement, catastrophizing, avoidance, sombreness and sleep.

**Results:**

Nine patients were included by six different primary care physiotherapists. Repeated measures on 196 data points showed that IPs Consequences, Personal control, Identity, Concern and Emotional response had a mediation effect on all three primary outcomes. The IP Personal control acted as a moderator for all primary outcomes, with clinically relevant improvements at 3 month follow up.

**Conclusion:**

Our study might indicate that some IPs have a mediating or a moderating effect on the outcome of a matched care physiotherapy treatment. Assessing Personal control at baseline, as a relevant moderator for the outcome prognosis of successful physiotherapy management of persistent low back pain, should be further eplored.

## Introduction

For decades now, low back pain (LBP) has been recognized as the main cause of years lived with disabilities [1]. Managing the global impact of LBP on patients, the increase of economic costs and the impact on society are challenging issues and therefore The Lancet Series on Low Back Pain 2018 included a call for action [2–5]. Management of persistent LBP has been proposed to shift from a unidimensional (focused on a patho-anatomical disorder) to a more holistic approach, making the transition from the biomedical model to a more biopsychosocial model [6–8]. Following this proposal, a physiotherapy treatment of LBP that incorporates biopsychosocial factors that play an important role in the patients’ LBP has the potential to increase the positive effect of physiotherapy. Examples of such treatment strategies are described in a Cochrane review on behavioral therapy for LBP; operant, cognitive-. and respondent strategies [9].

Most of the extensive body of knowledge on the management of LBP derives from systematic reviews and randomized controlled trials (RCTs). These designs represent the highest level of evidence in evidence based medicine. In addition, the randomized n-of-1 trials are also recognized as level 1 evidence in the Oxford Center for Evidence-Based Medicine 2011 levels of evidence [10, 11]. The use of evidence from systematic reviews and RCTs is a form of “reference class forecasting” and can be challenging for clinicians when making clinical relevant decisions for individual patients [12]. Does this patient fit within the “reference class” that has been reported to progress well with the intervention?. Recently, the call for a more personalized approach for LBP was made [13]. Such an approach could be a matched-care intervention, in which patients’ individual prognostic factors for recovery are assessed, and a response guided treatment package can be designed. A response guided treatment means that the treatment is matched to the ‘risk-profile’ of the patient. Known factors in such risk-profiles are psychological factors like fear of movement [14], catastrophizing [15], avoidance [16], somberness [17] and sleep [18]. It is hypothesized that such matched-care intervention may result in better treatment outcomes [19]. In this study we investigate the impact of taking into account another psychological factor in the risk-profile, namely Illness Perceptions’ (IPs), which is the core element of Leventhal’s Common Sense Model of health and Illness Representations (CSM) [20, 21].

The CSM is a parallel processing model that describes both cognitive and emotional representations of perceived health threats, leading to patients’ IPs resulting from these health threats. Higher IPs scores reflect a more threatening perception of illness and can be called ‘dysfunctional IPs’. These dysfunctional IPs may mediate or moderate persistent pain and disability [22] and personalizing management of LBP might involve addressing these IPs. Dysfunctional IPs have shown to attribute to higher pain intensity and lower physical functioning and quality of life in a variety of conditions [23]. It is not known how this attribution unfolds during a matched-care physiotherapy treatment, whether, for instance, IPs act as a mediator or moderator for LBP outcomes. A mediator indicates a part of the causal pathway. The intervention effect on the outcome goes through the mediator. A moderator on the other hand indicates that the intervention effect is different for different subgroups of the moderator [24]. This has not yet been researched in primary care physiotherapy, which is important in our health care system.

It is hypothesized IPs can mediate and/or moderate the association between intervention and outcome. To research the possible mediation and/or moderation effect of IPs on pain and disability, a multiple baseline Single Case Experimental Design (SCED) can be used to screen and measure patients’ individual prognostic factors for recovery before, during and after an intervention. In this study we use matched-care physiotherapy as the intervention for patients with persistent LBP and dysfunctional levels of IPs. In order to analyze the results from our experiment in this study, we pose the following three research questions:

1. Do pain intensity, physical function and pain interference change significantly during and after matched-care physiotherapy treatment?
2. Do Illness Perceptions mediate the effect of matched-care physiotherapy on pain intensity, physical function and pain interference?
3. Do baseline Illness Perceptions moderate the effect of matched-care physiotherapy on pain intensity, physical function and pain interference?

## Method

This study is designed according to The Single-Case Reporting Guideline In Behavioural Interventions (SCRIBE) checklist [25] and six primary care physiotherapy practices in The Netherlands participated. After a recruitement call on social media and within the professional network of the lead author (EdR), a group of physiotherapists signed up for a 2 day course, 6 hours/day. Within the course, the aim of the study, the design and lay-out of the matched-care intervention (treatment package see paragraph 2.3) were adressed. After this course, six eligible physiotherapists, each from different primary care physiotherapy practice, were included in the study after signing an informed consent. They had access to videos that summarized the discussed topics. The lead author was available at any time during the research period for support on the implementation of the project.

### Design

A multiple baseline SCED was applied. Participants completed repeated measurements during pre-treatment (phase A), during the treatment period (phase B) and a post-treatment period (phase A’). During all three phases of the study, the patients were asked to complete an online questionnaire (appendix C), twice a week in phase A and weekly in phases B and A’. Phase A acts as a control phase (no treatment given) for comparison with phases B and A’. The duration of phase A was 3 weeks with five to six measures. During phase B the patients received a matched-care treatment package (paragraph 2.3) by their physiotehrapist. The number of sessions was left to the discretion of the physiotehrapist, and therefore the duration of this phase varies accross patients. The content of the matched-care was response guided, meaning the intervention was based on the outcomes of the online questionnaires, which were administered by the patient the day before each consecutive intervention. The post-intervention period phase A’ took 12 weeks, independent of the duration of phase B. The study followed the guidelines of the declaration of Helsinki and the code of conduct for scientific research of our institute and was approved by the Medical Ethical Committee of the University of Applied Sciences, Utrecht (ref. no. 950002019).

### Patients

Eligible patients for this study were enrolled from six different primary care physiotherapy practices in The Netherlands within a period of 3 months. The invitation and treatment were performed by the same physiotherapist. Resulting from the design of the SCED, patients had to be willing to undertake phase A, which meant a 3 week wait while completing a total of five to six outcome measures before the first treatment in the clinic. We foresaw that this ‘waiting’ for a first treatment might be unattractive to patients and therefore of influence on the number of patients wanting to participate. This concern was addressed in a patient information letter by explaining the purpose of phase A; to determine a detailed baseline assessment which is important to design the match-care intervention. Inclusion criteria were age 18 years or older, LBP for at least 3-months, experiencing a movement problem in daily life due to LBP and having dysfunctional levels of at least one out of eight IP dimensions. Dysfunctional levels of IPs were based on a secondary analysis of an earlier study on the associations of IPs with patient burden with musculoskeletal pain [22] (Appendix A). We chose the fourth quartile as threshold (Table 1), expecting these high-level scores to represent dysfunctional IPs. When an eligible patient was identified at the clinic, a patient information letter was presented in which the study design was outlined. From there on, patients were free to choose whether to participate in the study, without any risk of being withheld from physiotherapy care.

**Table 1:** Dysfunctional illness perception threshold

	IP-dimension	Threshold
IP1	Consequences	8
IP2	Timeline	8
IP3	Personal control	7
IP4	Treatment control	4
IP5	Identity	8
IP6	Concern	8
IP7	Comprehensibility	5
IP8	Emotional	8

Exclusion criteria were specific LBP and existing (and diagnosed) psychiatric illness. When matching the inclusion criteria, patients were invited to participate by their physiotherapist after reading the patient information letter. Their decision on participating in the study did not have consequences for their treatment. After signing the informed consent, patients were included in the study.

### Matched-care treatment package

We used the Dutch guideline for LBP, and added a treatment package which was based on three frequently applied strategies for persistent LBP [9] (Appendix B). The specific aim of this response guided treatment package was to alter the dysfunctional levels of IPs by using cognitive, exposure and/or respondent strategies [9]. For instance, a cognitive strategy showed successful improvements in patient- relevant physical activities in patients with more than 1 year LBP [26]. Participating physiotherapists were asked to record the number of times each treatment strategy was applied during treatment phase B.

The treatment package offered the patient and physiotherapist the possibility to create a matched-care intervention as advised in the Dutch Guideline for Low Back Pain. This means that patients’ ‘risk-profile’ scores were assessed before each intervention and consequently these scores were used to design the response guided treatment, thereby providing matched-care (see paragraph 2.4).

### Measures

An online questionnaire was developed for assessing primary outcomes (pain intensity, physical function, and pain interference), secondary outcome (Illness Perceptions) and the co-variates (fear for damage/pain, pain anxiety, depressive mood, avoidance beliefs and sleep). Frequent administration allowed for monitoring the effect of the treatment package on all outcomes. These items are described below.

#### Primary outcome

Three outcome measures were chosen as primary outcome based on consensus recommendations from the literature; 1) pain intensity in the last 24-h [27]. 2) limitation in patients’ own selected physical function and 3) pain interference in daily activities [28]. All three primary outcome were assessed with an 11-point numeric rating scale (0–10). High scores for these three primary outcome measures mean respectively 1) higher levels of pain intensity, 2) stronger limitations in physical function and 3) greater interference of pain in daily activities. The physical function measure was adjusted to patients’ specific limitation in physical function (i.e. bending forward).

#### Illness perceptions secondary outcome

The Brief Illness Perception Questionnaire was used to assess patients’ Illness Perceptions representation on LBP [29, 30]. This questionnaire contains nine questions, of which the questions IP1 – IP8 were used in this study. Each item represents a different dimension of IPs. In order to ensure that all higher scores signify stronger dysfunctional IPs, data of the IP3–4 and IP7 were reversed before entering into the analyses.

#### Co-variates

The selection of co-variates was based on research showing these factors being associated with treatment outcome of LBP. They have also previously been used in a SCED study on persistent LBP [31]. The co-variates are: fear of movement [14], catastrophizing [15], avoidance [16], somberness [17] and sleep [18]. For all these co-variates we hypothesized that the higher their scores, the more negative impact they will have on the primary outcome.

#### Statistical analysis

To investigate whether primary outcomes change during and after matched-care physiotherapy treatment, linear mixed model analyses were performed, including all repeated measurements as outcome, and ‘phase’ as independent variables. First a crude analysis was performed. In a next analysis we controlled for the co-variates.

To investigate whether IPs mediate the effect of matched-care physiotherapy on primary outcomes, these adjusted analyses were performed including the IPs. Based on the change in the coefficient for treatment phase (two dummies, with phase A as reference category) the mediating role of each IP was evaluated independently. The magnitude of the mediation effect, the Indirect Effect, was calculated by subtracting the Direct Effect from the Total Effect.

Finally, to investigate whether baseline IPs moderate the effect of matched-care physiotherapy on primary outcomes, effect sizes were calculated for treatment phase and post-treatment phase (two dummies, with phase A as reference category) by adding the baseline IPs to the adjusted linear mixed models. The importance of the moderation was evaluated on significance (*p* < 0.05) of the interaction terms.

In addition to statistical significant effects, we evaluated the outcomes on their clinical meaningful effect using a threshold of ≥ 30% change in phase A’ on primary outcome from baseline scores phase A [32]. All analyses were performed with STATA® (version 15).

## Results

Table 2 presents the characteristics of participating physiotherapists. Six physiotherapists participated in the study, all working in different primary care physiotherapy practices across the Netherlands.

**Table 2:** Participating physiotherapists

Pht	Work setting	Years’ experience	Specialist	Particularities
I	Primary care	11	PSF	- ACT-trainer
II	Primary care	6	PSF^a^	- none
III	Primary care	4,5	MT^a^	- member pain network
IV	Primary care	4,5	PSF	- none
V	Primary care	35	MT	- Lecturer
VI	Primary care	34	MT	- Lecturer - EFIC pain physiotherapist

*Pht* participating physiotherapist, *MSc* Master of Science, *BSc* Bachelor of Science, *PSF* Psycho-Social Physiotherapy, *MT* Manual Therapy, *MMT* Master Manual Therapy, *ACT* Acceptance and Commitment Therapy

^a^ = student

Table 3 presents the characteristics of the nine participating patients, a sample size which was logistically a realistic achievement. Age ranged from 25 to 74 years. Reported baseline primary outcomes, mean (SD) were for Pain Intensity 5.6 (2.5), Physical Functioning 5.8 (2.7) and Pain Interference in Daily Life 5.9 (2.7). No adverse events were reported by the participating physiotherapists.

**Table 3:** Baseline scores participating patients, 44% female, age range 25–74

					Baseline Primary Outcome		
patient	Gender	Duration LBP (in weeks)	Oswestry (0–100)	Co-morbidity	PI	PF	PIDL
1	Male	> 500	70	Heart condition	8	6	8
2	Male	15	52	–	7	8	8
3	Female	12	38	–	3	2	2
4	Male	> 250	70	Rheumatoid arthritis	7	8	9
5	Male	> 150	42	–	7	9	8
6	Female	32	80	Rheumatoid arthritis	9	8	8
7	Female	> 200	32	–	7	9	7
8	Male	12	24	Osteoarthritis	2	5	1
9	Female	52	38	PCOS. Hashimoto	3	6	6

*PI* Pain Intensity, *PF* Physical Functioning, *PIDL* Pain Interference in Daily Life.

Table 4 shows which baseline IPs dimensions reached the threshold score, as one of the inclusion criteria, per patient.

**Table 4:**
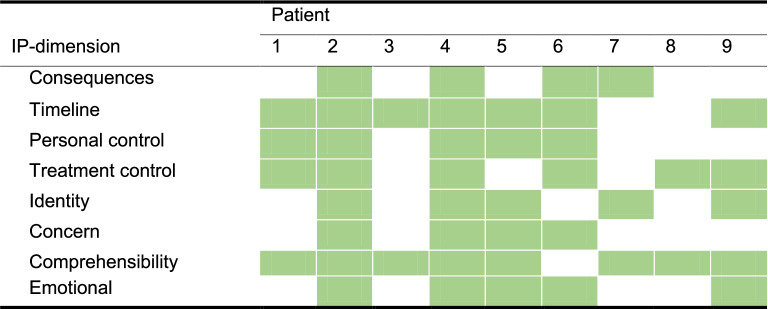
IPs dimension inclusion criteria per patients’ exceeded threshold

In Table 5, a synthesis of the applied treatment packages is reported. The duration average of phase B was 8 weeks, with a minimum of 3 weeks and a maximum of 15 weeks. The number of treatment sessions varied from 3 to 10. Participating physiotherapists applied a combination of treatments strategies, as described in appendix B, within one treatment session. The cognitive strategy was the most frequently reported strategy.

**Table 5:** Duration phase B and synthesis of interventions per participating patient

			Treatment strategy^a^		
patient	Duration phase B (in weeks)	Number of treatments	Cognitive strategy	Operant strategy	Classical conditioning
1	6	7	6	2	2
2	15	9			
3	8	5	5	4	4
4	9	5	3	1	4
5	8	7	5	4	3
6	8	5	5	2	2
7	7	10	9	5	6
8	8	6	4	3	4
9	3	3	3	1	2

^a^ Number of times each treatment strategy was applied during treatment phase B, self-reported by physiotherapist

Table 6 shows the results of the linear mixed model analyses to investigate whether primary outcomes changed during and after matched-care physiotherapy. During treatment, all three outcomes show a significant and clinical meaningful improvement of ≥ 30% effect. The adjusted effects shows clinical meaningful improvement of ≥ 30% for pain and physical functioning. Post treatment, the effect did not wash-out. Remaining in significant and clinical meaningful improvement of ≥ 30% for all three outcomes.

**Table 6:** Final linear mixed model Regression effects, study phase A as reference class

	During treatment				Post treatment			
	Effect crude	95% CI	Effect adjusted^b^	95% CI	Effect crude	95% CI	Effect adjusted^b^	95% CI
Pain Intensity	-2.23^a^	−2.91 / -1.54	−1.3	− 1.9 / -0.7	−3.52^a^	−4.21 / -2.84	− 1.8^a^	− 2.4 / -1.2
Physical Functioning	− 2.41^a^	− 3.07 / − 1.76	-1.6^a^	− 2.2 / -1.1	− 4.10^a^	− 4.50 / -3.44	− 2.6^a^	− 3.2 / -1.1
Pain Interference Daily Life	− 2.39^a^	− 3.05 / -1.73	−1.3	− 1.9 / 0.7	− 4.21^a^	−4.87 / -3.55	−2.4^a^	− 3.0 / -1.8

*SD* Standard deviation, All outcome = *P <* .05, ^a^ = Clinical meaningful improvement ≥ 30% baseline score [32].

^b^adjusted for: fear of movement, catastrophizing, avoidance, somberness and sleep

Table 7 shows the results of the mediation analyses performed on the adjusted models. Five of the 8 IP dimensions substantially mediated the total effect on all three primary outcomes. For instance, the IP dimension Consequences mediated for 38.5% the effect of the treatment on pain intensity during the treatment (Phase B) and this increased to 38.9% for the post-treatment (Phase A’). The IP Consequences and Identity were strong mediators in all three primary outcomes. The other dimensions that mediated the effect of the treatement on the outcome were Identity, Concern, Emotional and Personal control. Three IPs showed lesser mediation effects, with Timeline being the smallest mediator by 1.7% for Physical functioning post treatment.

**Table 7:** Results of the analyses to evaluate the mediating influence of IPs on treatment adjusted effect on primary outcomes

	Total adjusted effect of treatment on primary outcomes											
	Pain Intensity				Physical functioning				Pain interference daily life			
	During Treatment − 1.3 (CI − 1.9 / -0.7)		Post Treatment − 1.8 (CI − 2.4 / -1.2) ^a^		During Treatment − 1.6 (− 2.2 / -1.1) ^a^		Post Treatment −2.6 (CI − 32 / -1.1) ^a^		During Treatment −1.3 (CI-1.9 / 0.7)		Post Treatment − 2.4 (CI − 3.0 / -1.8) ^a^	
Indirect Effect (mediation) of Illness Perception Dimension	IE	%	IE	%	IE	%	IE	%	IE	%	IE	%
Consequences	−0.5	38.5	−0.7	38.9	−0.5	31.3	−1.2	46.2	−0.6	46.2	−1.3	54.2
Timeline	0.0	0.0	−0.1	5.6	0.0	0.0	0.0	0.0	0.0	0.0	−0.1	4.2
Personal Control	− 0.2	15.4	− 0.2	11.1	− 0.2	12.5	− 03	11.5	− 0.1	7.7	− 0.2	8.3
Treatment Control	−0.1	7.8	−0.1	5.6	−0.1	6.3	0.0	0.0	−0.1	7.7	0.0	0.0
Identity	−0.5	39.5	−0.7	38.9	−0.5	31.3	−1.2	46.2	−0.7	53.8	−1.5	62.5
Concern	−0.4	30.8	−0.2	11.1	−0.5	31.3	−0.8	30.8	−0.4	30.8	−0.8	33.3
Comprehensibility	−0.1	7.8	−0.1	5.6	−0.1	6.3	−0.1	3.8	−0.1	7.7	−0.1	4.2
Emotional	−0.2	15.4	−0.7	38.9	−0.1	6.3	−0.6	23.1	−0.2	15.4	−0.8	33.3

*CI* 95% Confidence Interval, ^a^ = Clinical meaningful improvement ≥ 30% baseline score [32], *IE* Indirect Effect (Mediation Effect), % = Percentage mediation.

Table 8 shows the statistically significant results of the moderation analyses performed on the adjusted models. The IPs dimension Personal control moderated the treatment effects for all three primary outcomes. There is a stronger treatment effect for patients with a low baseline score (0–7) on Personal control versus patients with high baseline scores (8–10) on Personal control. This means that when patients experienced higher control (0–7) over their condition at baseline, the stronger the positive effect on the primary outcome was in both the treatment and the post-treatment phases.

**Table 8:** Final linear mixed model effects for IPs as moderator for Primary Outcomes with Study phase A as reference class, adjusted for co-variates

	Pain Intensity				Physical functioning				Pain interference daily life			
	During Treatment		Post Treatment		During Treatment		Post Treatment		During Treatment		Post Treatment	
Illness Perception	TE	CI	TE	CI	TE	CI	TE	CI	TE	CI	TE	CI
Personal control												
Low baseline score (0–7) *n* = 140	−2.1^a^	−2.9 / -1.2	−2.7^a^	−3.5 / -1.8	−2.1^a^	−2.9 / -1.2	−3.3^a^	−4.2 / −2.6	-2.1^a^	− 3.0 / -1.3	− 3.7^a^	−4.5 / -2.8
High baseline score (8–10) *n* = 56	−0.8	−1.5 / -0.1	−1.3	− 2.0 / -0.5	−1.3	− 2.0 / -0.7	−2.1^a^	− 2.8 / -1.4	−0.8	− 1.5 / -0.1	− 1.6	−2.3 / -0.9
Treatment control												
Low baseline score (0–4) *n* = 127					−2.1^a^	− 2.8 / -1.4	−2.9^a^	− 3.6 / -2.2				
High baseline score (5–10) *n* = 69					−1.0	−1.8 / -0.2	−2.3^a^	−3.1 / -1.5				
Identity												
Low baseline score (0–8) *n* = 144									−2.0^a^	−2.8 / -1.2	2.8^a^	−3.6 / -2.0
High baseline score (9–10) *n* = 52									−0.7	−1.5 / 0.1	2.1^a^	−3.0 / − 1.3
Concern												
Low baseline score (0–8) *n* = 153									-1.8^a^	−2.5 / -1.0	2.6^a^	−3.2 / -1.9
High baseline score (9–10) *n* = 43									−0.8	−1.6 / 0.1	2.3^a^	−3.2 / -1.4
Emotional response												
Low baseline score (0–8) *n* = 145									−2.0^a^	−2.8 / -1.2	2.8^a^	−3.6 / 2.0
High baseline score (9–10) *n* = 51									−0.7	−1.5 / 0.1	2.1^a^	−3.0 / -1.3

A = pre-treatment, B = during treatment, A’ = post-treatment, TE = Total Effect, All outcome = *P <* .01, ^a^ = Clinical meaningful improvement ≥ 30% baseline score [32].

The IPs dimension Treatment control showed a moderating effect for Physical functioning. This indicates a stronger treatment effect for patients with a low baseline score (0–4) on Treatment control versus patients with high baseline scores (5–10) on Treatment control. This means that the more patients expected treatment to control their condition at baseline, the stronger the effect on the primary outcome was in both the treatment phase B and the post-treatment phase A’.

For Pain Interference in Daily Life, baseline low scores in the IPs dimensions Identity (0–8), Concern (0–8) and Emotional response (0–8) showed stronger effects for both treatment and post-treatment phase versus patients with high baseline scores.

The moderating effect of the IPs dimensions Personal Control, Identity, Concern and Emotional response did not wash out during the post treatment phase.

## Discussion

In this matched-care physiotherapy treatment for patients with persistent LBP SCED-study, we showed a statistically significant and clinically meaningful improvement in decreasing pain intensity, increased physical function and lesser pain interference in daily life during and 3 months post-treatment. We did not observe a wash-out phenomenon during the post treatment phase. Furthermore, we found five IP dimensions mediating the effect on all three primary outcomes; namely, Consequences (45.2–56.3) Personal control (8.1–15.7), Identity (46.7–52.9), Concern (15.6–34.3) and Emotional response (24.3–38.9). At baseline, the IP Personal control acted as a moderator for all primary outcomes. In the post treatment phase the IPs Personal Control, Identity, Concern and Emotional response also acted as moderator.

### Illness perceptions as mediator

The search for causal mechanisms for non-specific LBP has been a quest for decades now [33, 34]. Identifying such mechanisms is useful, for instance, when designing a ‘Magic Bullet’ cure, for a condition that is primarily caused by a pathoanatomical impairment [35]. In the case of persistent musculoskeletal pain like LBP, such pathoanatomical impairment most likely cannot be identified. LBP is considered to be a symptom of a complex condition with multiple contributors to both pain and associated limitations in physical function, including psychological factors, social factors, biophysical factors, comorbidities, and pain-processing mechanisms [4]. Models for management of complex conditions should incorporate these multiple contributors, including patients’ beliefs about their condition [35, 36]. IPs are thought of as one aspect of these beliefs [36]. Through mediation analyses we identified five IP dimensions that mediated the total effect of our matched-care physiotherapy treatment package [17]. Intervention studies on how to alter IPs in LBP are scarce. We know of one RCT that looked at altering baseline IPs with cognitive treatment to improve patient relevant physical activities [26]. In this study IP dimensions Timeline cyclical, Consequences, Personal control and Coherence attributed 14.4% of the explained variance to physical activities. This partly overlaps with our results. We found IP dimensions Consequences and Personal control also significantly mediating the total effect on all three primary outcomes. The effects in our study are found within a non-controlled design and should be further tested in a larger population and with a different design such as a randomized controlled trial.

### Illness perceptions as moderator

The course and prognosis of developing persistent LBP have been extensively researched [37]. The overall findings are reported as; “Low to moderate levels of pain and disability were still present at one year, especially in the cohorts with persistent pain.” In a Cochrane review on individual recovery expectations it is concluded: “Our findings suggest that recovery expectations should be considered in future studies, to improve prognosis and management of low back pain” [38]. We found the IP dimension Personal control to be moderating the effect on all three primary outcomes. This IP dimension can be seen as reflecting patients’ expectations about the effect of the treatment. We therefore would like to advise to consider the IP Personal control in future research concerning treatment and prognosis of LBP.

### Study imitations

Several limitations need to be considered. First, there was no randomization. The effects in our study are found within a non-controlled design. We explicitly focused on a ‘matched care intervention’. Meaning that the intervention was tailored on the patients’ clinical presentation, and therefore randomization was not included in our design. Secondly, selection bias of patients. The patients were selected by the participating physiotherapists, therefore the generalizability of our results is somewhat limited. Thirdly, patients were required to complete a questionnaire, monitoring their progress on a weekly basis for several months. This may have given rise to the awareness of being studied. This possibly impacted behavior [39], resulting in a Hawthorne effect.

Fourthly, there is a potential sampling bias of treating / participating physiotherapists due to the use of convenience sampling of physiotherapists via social media and within the network of the first author. They were invited to our two-day course to be informed on the design of the study. These physiotherapists might not be representative of the physiotherapy community in the Netherlands. Fifthly, we do not have data to analyze the treatment fidelity of participating physiotherapists on delivering the matched-care treatment package. The weight this has on the effects is not clear. We tried to minimize this limitation by including several implementation interventions addressing fidelity of the physiotherapists to participate in the study: a 2 day course, videos were accessible demonstrating how to apply treatment strategies and the use of repeated measures during the treatment phase.

Finally, due to the design of this study conclusions about causal relations between IPs and the primary outcome cannot be drawn. Further studies on the temporal order of the associations between matched-care physiotherapy, IPs and treatment outcomes are recommended.

### Study strengths

There are several strengths of this study to be considered. First, the use of repeated measures and a matched-care intervention instead of a strict treatment protocol allowed the physiotherapists to adjust their interventions to the clinical status of the patient with each new appointment. This dynamic and cyclical process is commonly used by physiotherapists and is a reflection of their clinical reasoning process [40], making this design representative for daily practice. For example, if the patient shows a sufficient decrease of safety behaviors, than withdrawal of safety behavior strategy is justified [41]. Secondly, within the model of Illness Representations by Leventhal it is hypothesized that dysfunctional perceptions affect pain and limitations in physical functioning. The use of an IP threshold as an inclusion criterion implies good diagnostics for creating a window of opportunity to improve pain and physical functioning by altering IPs. Thirdly, this study is a good example of how to include physiotherapists’ clinical relevant decisions for avoiding problems concerning “reference class forecasting”. Such forecasting relies on prediction from past reference classes, a model which may not be the most suitable because of the large variability in clinical signs and symptoms in patients with low back pain. In our study we explicitly incorporated psycho-social elements which were relevant for that patient as was shown in their ‘risk-profile’.

### Practical implications

The use of a matched-care physiotherapy treatment is accompanied by a decrease of pain and physical function related health problems in patients with persistent low back pain. This type of research, looking at treaments that incorporate a dynamic and cyclical process is a reproduction of daily physiotherapy practice. We would like to encourage this way of working and researching the effectiveness of physiotherapy.

In earlier research, we concluded based on a longitudinal study with two timepoints that baseline IPs did not predict poor recovery on pain and/or physical function after three. The results of this study are not in line with these findings. For instance, dysfunctional baseline IP Personal control scores (7–10) might be relevant as a moderating factor, meaning that physiotherapists could consider to use item 3 of the Brief IPQ-DLV for the baseline assessment of patients’ perceptions on controllability of their condition. This should be further inverstigated. A specific intervention targeting such a dysfunctional perception might than be appropriate. Further, evaluating the change in the IPs dimension Consequences, Personal control, Identity, Concern and Emotional response during treatment might be relevant because our results showed a mediating effect of change in these perceptions. Though further explorations are needed, if one of these perceptions does not change during treatment there might still be room for improvement by specifically targeting these perceptions with interventions. Thereby, applying the principles of matched-care treatment.

## Conclusion

Our study might indicate that some IPs have a mediating or a moderating effect on pain intensity, physical function and pain interference during a matched care physiotherapy treatment.

Our findings indicate that the IP dimensions Consequences, Personal control, Identity, Concern and Emotional response, might be important to include in a matched-care treatment of LBP, because they enhance the positive mediation effect of all three primary outcomes. In addition, assessing Personal control at baseline, as a relevant moderator for the outcome prognosis of successful physiotherapy management of persistent low back pain, should be further eplored.

## Data Availability

the datasets used and/or analysed during the current study are available from the corresponding author on reasonable request. The datasets are stored in a repository of the University of applied sciences Utrecht, which can be accessed from a University account.

## Appendix A

IP 1 How much does your illness effect your life?

IP 2 How long do you think your illness will continue?

IP 3 How much control do you feel you have over your illness?

IP 4 How much do you think your treatment can help your illness?

Q1 = 1st quartile, Q4 = 4th Quartile, PI = Pain Intensity last 24 h, PSFS = Patient Specific Functioning Scale, 4DSQ = Four-Dimensional Symptom Questionnaire.

IP 5 How much do you experience symptoms from your illness?

IP 6 How concerned are you about your illness?

IP 7 How well do you feel you understand your illness?

IP 8 How much does your illness affect you emotionally? (e.g. does it make you angry. Scared. upset or depressed?

Q1 = 1st quartile, Q4 = 4th Quartile, PI = Pain Intensity last 24 h, PSFS = Patient Specific Functioning Scale, 4DSQ = Four-Dimensional Symptom Questionnaire.

## Appendix B

### The matched-care treament of the Single-Case Experimental Design

#### Intervention

[The intervention is based on usual care following the low back pain guideline of the Royal Dutch Physiotherapy Association [16] and will target patients whom are classified in ‘patient profile 3’. This means that this study includes patients that have an abnormal course with dominant presence of psychosocial factors impeding recovery.

The intervention is considered to be delivered as proposed in the guideline, with an additional matched-care treatment package. This package focusses on patients’ specific Illness Perception (IPs) regarding his or her low back pain. This means if IPs are considered to be dysfunctional before and during treatment, these IPs will be seen as prognostic factor for poor recovery of pain intensity and physical function. The aim is to alter dysfunctional IPs to more functional perceptions by the advised strategies for consistent (back) pain [5, 8, 15, 17]. These cognitive, exposure and respondent strategies will be response guided at the beginning and during each intervention session.

The additional treatment package will be matched with the scores of the IPs before each treatment session. Patients whose score are within the 4th-quartile range (Table 1), are seen as indicative for dysfunctional IPs, will be challenged to rethink their perception by a combination of the three proposed strategies. This means that the physiotherapist together with the patient must decide on which strategy to start with and when to switch to another strategy. This decision-making process is an essential part of the intervention and will be shaped by shared decision-making [2] and can be seen as a response guided intervention. This treatment approach can be seen more as reflective than as descriptive. Meaning the patient guides her or his own meaningful and safe strategies to cope with their pain condition. The physiotherapist is more a reflective, instead of a problem-solving practitioner. For full description of the treatment package.

**Table:**

	Treatment strategies			
	Education [10]	Exposure [7]	Graded activity [4, 11]	Safety behavior [6]
Underlying paradigm	Cognitive strategy	Cognitive strategy	Operant strategy	Classical conditioning
Treatment aim	Increase level of pain understanding	Decrease fear related disability	Increase physical resilience	Reducing safety behaviour

#### Treatment package

Each strategy within the treatment package consists of a diagnostic- and a treatment phase. The diagnostic-phase determines if the strategy is indicated to be used and if so, the treatment phase will then deliver the treatment as intended within this specific strategy.

- The cognitive-based strategy

Pain neuroscience education has been proven to be useful for reducing pain, improving patient knowledge of pain, improving function and lowering disability, reducing psychosocial factors, enhancing movement, and minimizing healthcare utilization [9].

- Diagnostic-phase

The revised neuro physiology pain questionnaire will be used for assessing patients’ baseline knowledge of pain physiology [1]. The outcome of this questionnaire, together with The Brief-IPQ-DLV baseline scores will be determining the content of the treatment phase.

- Treatment phase

By a number of tools, the patients’ knowledge and perceptions about their pain condition will be discussed. Important part of the intervention will be pain neuroscience education. Main message will be that pain mainly is about being a symptom that is formed from past experiences, sensory input and contextual circumstances [14], not about tissue damage alone.

- The Operant-based strategy

Is based on the Operant Learning Theory (OLT) introduced by Fordyce for managing chronic pain [3]. The use of OLT has been shown to be useful [4], treatment is advised to be customized to the bio-psych-social needs of the patient [12].

- Diagnostic-phase

The Phoda will be used to rate the level of patients’ fear related avoidance of daily activities. The outcome of this method, 3–5 most highly feared daily activities, together with The Brief-IPQ-DLV baseline scores will be selected to be expose the feared activities with movement/exercise related OLT.

- Treatment phase

Exposure with movement will be used to adjust patients’ fear and beliefs about the harmfulness of the daily activity. There will be no upfront defined route of ‘graded exposure’ before the treatment session. The start of the exposure will always be aimed on the least feared activity first but might be directly followed with the most feared activity, depending on the pace in which patients’ fear and beliefs are responding.

- The respondent-based strategy

Is based on safety behaviour expression, such as propping with hands and avoiding loading painful body part [13].

- Diagnostic-phase

The diagnostics is primarily done via observation by the physiotherapist during interview, examination and treatment. These observations will focus on safety and communication behaviors and sympathetic responses.

- Treatment phase

Cited from O’sullivan 2018: *“These observations then form the basis of a series of guided behavioral experiments. These guided experiments explicitly seek to reduce sympathetic responses and abolish safety and communicative behaviors (via relaxed diaphragmatic breathing, body relaxation, awareness, and control), prior to and while gradually exposing individuals to their feared, avoided, and painful tasks.”*

##### References appendix B

1. Catley MJ, O’Connell NE, Moseley GL. How Good Is the Neurophysiology of Pain Questionnaire? A Rasch Analysis of Psychometric Properties. *The Journal of Pain*. 2013;14(8):818–827. doi:10.1016/j.jpain.2013.02.008.

2. Epstein RM, Alper BS, Quill TE. Communicating evidence for participatory decision making. *Jama: The Journal of the American Medical Association*. 2004;291(19):2359–2366. doi:10.1001/jama.291.19.2359.

3. Fordyce WE. Behavioral factors in pain. *Neurosurg Clin N Am*. 1991;2(4):749–759.

4. Gatzounis R, Schrooten MGS, Crombez G, Vlaeyen JWS. Operant Learning Theory in Pain and Chronic Pain Rehabilitation. *Curr Pain Headache Rep*. 2012;16(2):117–126. doi:10.1007/s11916-012-0247-1.

5. Henschke N, Ostelo RW, van Tulder MW, et al. Behavioural treatment for chronic low-back pain. *Cochrane Database Syst Rev*. 2010;(7):CD002014. doi:10.1002/14651858.CD002014.pub3.

6. Hoffman LJ, Chu BC. When Is Seeking Safety Functional? Taking a Pragmatic Approach to Distinguishing Coping From Safety. *Cognitive and Behavioral Practice*. 2019;26(1):176–185. doi:10.1016/j.cbpra.2018.11.002.

7. Hollander den M, Goossens M, de Jong J, et al. Expose or protect? A randomized controlled trial of exposure in vivo vs pain-contingent treatment as usual in patients with complex regional pain syndrome type 1. *Pain*. 2016;157(10):2318–2329. doi:10.1097/j.pain.0000000000000651.

8. Jensen M. Toward the development of a motivational model of pain self-management. *The Journal of Pain*. 2003;4(9):477–492. doi:10.1016/S1526-5900(03)00779-X.

9. Louw A, Diener I, Butler DS, Puentedura EJ. The Effect of Neuroscience Education on Pain, Disability, Anxiety, and Stress in Chronic Musculoskeletal Pain. *Archives of Physical Medicine and Rehabilitation*. 2011;92(12):2041–2056. doi:10.1016/j.apmr.2011.07.198.

10. Moseley GL, Butler DS. Fifteen Years of Explaining Pain: The Past, Present, and Future. *The Journal of Pain*. 2015;16(9):807–813. doi:10.1016/j.jpain.2015.05.005.

11. Moseley GL, Vlaeyen JWS. Beyond nociception. *Pain*. 2015;156(1):35–38. doi:10.1016/j.pain.0000000000000014.

12. Nielson WR, Weir R. Biopsychosocial approaches to the treatment of chronic pain. *The Clinical Journal of Pain*. 2001;17(4 Suppl):S114-S127.

13. O’Sullivan PB, Caneiro JP, O’Keeffe M, et al. Cognitive Functional Therapy: An Integrated Behavioral Approach for the Targeted Management of Disabling Low Back Pain. *physical therapy*. 2018;98(5):408–423. doi:10.1093/ptj/pzy022.

14. Ongaro G, Kaptchuk TJ. Symptom perception, placebo effects, and the Bayesian brain. *Pain*. 2019;160(1):1–4. doi:10.1097/j.pain.0000000000001367.

15. Rudy TE, Kerns RD, Turk DC. Chronic pain and depression: toward a cognitive-behavioral mediation model. *Pain*. 1988;35(2):129–140.

16. Staal JB, Hendriks EJM, Heijmans M, et al. KNGF Guideline Low back pain. January 2013:1–7.

17. Vlaeyen JW, Haazen IW, Schuerman JA, Kole-Snijders AM, van Eek H. Behavioural rehabilitation of chronic low back pain: comparison of an operant treatment, an operant-cognitive treatment and an operant-respondent treatment. *Br J Clin Psychol*. 1995;34 (Pt 1):95–118.

## Appendix C

Online questionnaire to assess primary outcomes, Illness Perceptions and co-variates. All items scored on 11-point scale (0–10) and anchored by words appropriately related to each question. Outcome score were reversed to lower score meaning less dysfunction.

Primary outcome.

- What was the average back pain over the past 24 h?
- In the past week, how difficult was it to perform your self-proclaimed activity?
- How much has the back pain limited you in your daily activities?

Illness Perceptions secondary outcome

- How much does your illness affect your life?
- How long do you think your illness will continue?
- How much control do you feel you have over your illness?
- How much do you think your treatment can help your illness?
- How much do you experience symptoms from your illness?
- How concerned are you about your illness?
- How well do you feel you understand your illness?
- How much does your illness affect you emotionally? (e.g. does it make you angry, scared, upset or depressed?)

Co-variates

- My pain complaints will decrease if I were to exercise.
- When I am in pain, I wonder whether something serious may happen.
- I avoid important activities when I hurt.
- How much have you been bothered by feeling depressed in the last 24-h?
- I can sleep at night.
